# MAAR-Net: Multi-scale attention-assisted residual neural network for renal microvascular structure segmentation

**DOI:** 10.1371/journal.pone.0342752

**Published:** 2026-03-04

**Authors:** Tingting Wang, Baoguang Lin, Tong Jiang, Hengjiao Wang, Defu Yang, Feng Shang, Long Li, Ying Li, Mengyan Zhao, Ying Xu, Ying Yan

**Affiliations:** 1 Department of Radiationtherapy, General Hospital of Northern Theater Command, Shenyang, Liaoning, China; 2 Department of Oncology, General Hospital of Northern Theater Command, Shenyang, Liaoning, China; Shijiazhuang Tiedao University, CHINA

## Abstract

Renal disease represents a significant public health concern, with renal microvascular lesions playing a crucial role in disease progression. Accurate segmentation of this microvasculature is therefore essential for precise pathologic evaluation. While deep learning offers substantial opportunities in medical image segmentation, the complex structure of renal microvessels poses a considerable challenge. Existing models often struggle to achieve high segmentation accuracy while maintaining branch continuity, suppressing background interference, and delineating tissue boundaries. To address these challenges, we propose a novel deep learning architecture termed the Multiscale Attention-Assisted Residual Neural Network (MAAR-Net). Built upon a U-Net encoder-decoder backbone, MAAR-Net integrates multiscale residual blocks and a high-semantic feature extraction layer to expand the receptive field and enrich semantic information. Depth-separable convolutional attention blocks are incorporated into skip connections to enhance the capture of global and local features, thereby refining segmentation performance. Additional segmentation branches are included to aggregate multi-receptive-field information, further improving segmentation efficiency. Our experiments, conducted on the HuBMAP dataset of 2D PAS-stained kidney histology images, demonstrate the effectiveness of MAAR-Net. The model achieves an Intersection over Union (IoU) of 0.5063 and an F1-score of 0.6751, outperforming other mainstream segmentation models. To facilitate clinical deployment, the optimized model is subsequently compressed via structured pruning to reduce size and increase speed, followed by quantification to lower computational resource consumption. These optimizations ensure the model’s suitability for real-time performance in practical diagnostic applications, independent of dedicated workstations or cloud servers. The results collectively validate the robustness and practical utility of our approach for accurate renal microvessel segmentation in real-world scenarios.

## 1. Introduction

### 1.1. Research background and main work

Kidney disease represents a major and underrecognized global public health challenge, serving as both a direct cause of mortality and a significant risk factor for cardiovascular disease [1]. In 2017, chronic kidney disease (CKD) was the 12th leading cause of death worldwide, with cardiovascular mortality attributable to reduced renal function accounting for 4.6% of all global deaths [2]. The World Health Organization has since reported CKD among the top ten causes of mortality [3]. The prevalence is substantial; in the United States, over 15% of adults were affected by 2021 [4], and an estimated 14% of the population had CKD in 2023, of whom up to 90% were unaware of their condition [5].

Major risk factors for CKD include hypertension and diabetes, which are highly prevalent in aging populations [6]. Data from China underscore this burden, indicating that 90% of an estimated 82 million individuals with CKD are undiagnosed, with hypertension and diabetes present as comorbidities in 60.5% and 31.3% of cases, respectively [4]. The frequently asymptomatic and protracted early course of CKD leads to underdiagnosis, which delays treatment, increases future health risks [7,8], elevates long-term healthcare costs, and impairs patient quality of life, creating substantial personal and societal burdens.

Therefore, reliable tools for early detection and assessment are critically needed. Medical image analysis, particularly precise segmentation of renal microvascular structures, is essential for improving diagnosis. In this work, we propose a Multi-scale Attention-Aided Residual Neural Network (MAAR-Net) for the segmentation of renal microvascular structures. The model is trained and evaluated using 2D PAS-stained whole-kidney histology images from the HuBMAP dataset. This approach aims to provide a robust tool for quantitative histological analysis, with significant potential clinical utility. The primary contributions of this study are summarized as follows:

(1) To enhance feature representation for complex microvasculature, we introduce a modified encoder built upon improved BasicBlock modules. This design enlarges the model’s receptive field, enriches semantic information capture, and mitigates issues of network degradation and gradient vanishing, leading to more robust feature extraction for intricate renal microvascular structures.
(2) To address the challenge of segmenting small, low-contrast microvessels, we propose and integrate a depthwise separable convolutional attention mechanism within the U-Net’s skip connections. This mechanism enables the network to dynamically focus on salient vascular features while suppressing irrelevant background information from both channel and spatial dimensions, thereby improving segmentation accuracy.
(3) To leverage multi-scale information and improve model robustness, we incorporate auxiliary segmentation branches during training. This strategy allows the network to effectively aggregate features from multiple receptive fields, enhancing its ability to capture vascular structures at varying scales.

### 1.2. Structure of the paper

The remainder of this paper is organized as follows. Section 2 reviews related work on medical image segmentation. Section 3 details the proposed MAAR-Net architecture, including its core modules and the subsequent model compression strategies involving structured pruning and quantification. Section 4 presents the experimental results and analysis, comprising performance comparisons, ablation studies, and validation of the compression efficacy. Section 5 concludes the paper by summarizing the entire work and its contributions. Finally, Section 6 outlines potential directions for future research.

## 2. Related work

Medical image segmentation is a computer vision-based technique that divides images by identifying the category of each pixel. Using machine vision, it automatically separates target regions to extract spatial information, such as for tissues, organs, or vascular structures. This process is essential for qualitative analysis in medical imaging [9,10] and supports diagnostic work for medical professionals.

Early medical image segmentation relied on conventional techniques rooted in image processing. These methods include several categories. Edge detection-based segmentation classifies and locates sharp discontinuous pixels; for instance, Xie et al. [11] used a distance regularized level set technique to segment nasal cavity boundaries. Threshold-based segmentation selects one or more thresholds to classify pixels, as seen in Varga A et al.‘s [12] semi-automatic cardiac MRI segmentation method. Region-based segmentation groups areas with similar features like color or luminance, employing algorithms such as region growing and split-and-merge. Dai et al. [13], for example, applied 3D region growing to isolate trachea and bronchial tissues, followed by convex hull optimization. Graph theory-based segmentation defines boundaries to partition images into subgraphs, using algorithms like GraphCut [14] and GrabCut [15]. Wu et al. [16] enhanced GrabCut for 3D breast ultrasound segmentation by incorporating polygonal interactions and a grayscale Gaussian mixture model.

Researchers in machine vision also develop segmentation algorithms by integrating specific theories. Representative approaches include those based on fuzzy set theory and wavelet transform with automatic threshold selection. Hua et al. [17], for example, applied an enhanced fuzzy clustering technique to brain MRI segmentation, using the image histogram as the clustering center and adjusting iterations to improve results.

Deep learning-based image segmentation is highly valued for its superior learning ability and performance compared to traditional methods. In medical image segmentation, deep learning has become the industry standard [18]. Fully supervised learning is the most common technique, depending on data volume and label accuracy; machine learning broadly includes semi-supervised, unsupervised, and fully supervised learning [19]. Recently, many scientists have applied deep learning to medical tasks. The development of Fully Convolutional Networks (FCN) [20] significantly advanced semantic segmentation. Following FCN’s success, researchers have adapted it for medical use: Ben-Cohen et al. [21] used FCN for liver segmentation in CT images; Avijit Dasgupta et al. [22] modeled retinal blood vessel segmentation as a multi-label inference problem with a joint loss function in an FCN architecture; Fausto Milletari et al. [23] introduced a dice coefficient-based loss function and 3D convolutional network for MRI prostate segmentation; Nie et al. [24] developed an N-shaped dense FCN for thyroid nodule segmentation, proposing a stackable dilated convolutional block to recover lost semantic features; Chaitanya Kaul et al. [25] integrated attention mechanisms into a ResNet [26] and SE [27] hybrid network for lung lesion and skin cancer segmentation, achieving competitive performance.

Based on FCN, researchers have created other semantic segmentation networks like DeepLab [28], SegNet [29,30], and BiseNet [31], advancing image segmentation technology. Sha et al. [32] proposed the ZHPO-LightXBoost model for pesticide residue prediction and verified and tested it on four independently constructed datasets. Although these models offer good speed and accuracy, their performance in medical image segmentation is suboptimal due to differing complexity and precision requirements compared to natural images. Currently, medical image segmentation primarily relies on U-Net and its variants, which employ an encoder-decoder structure. Ronneberger et al. [33] proposed U-Net in 2015, featuring a U-shaped encoder-decoder network with skip connections that merge features during up-sampling, yielding excellent results.

U-Net has inspired many improved networks. Zhou et al. [34] introduced U-Net++ to address gradient vanishing through dense skip connections and deep supervision on feature maps, with pruning during inference to reduce time. Huang et al. [35] developed U-Net3+ to enhance boundary accuracy and reduce over-segmentation, using full-scale skip connections and a hybrid loss function for multi-scale feature fusion. Dinh et al. [36] proposed U-Lite, a lightweight U-Net that applies axial depthwise convolution in the encoder-decoder and multiple 3x3 axial dilated depthwise convolutions in the bottleneck module, cutting parameters while maintaining accuracy. Yuan et al. [37] proposed the KAU-Net model to achieve the reuse and re-exploration of historical information through the information interaction between the image feature path and the historical information path, enabling the deep layer to learn comprehensive features. Sha et al. [38] proposed the SSC-Net model for tongue segmentation and multi-label classification.

## 3. Methods

**Fig 1:**
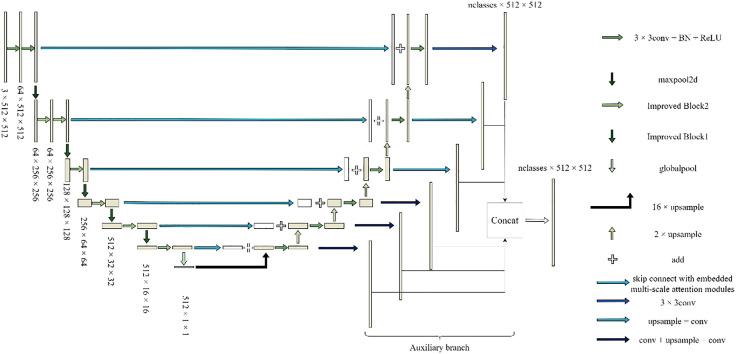
The overall structure of the MAAR-Net model.

**Fig 2:**
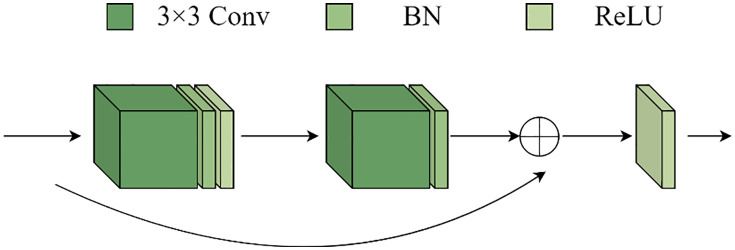
The original BasicBlock1 structure.

**Fig 3:**
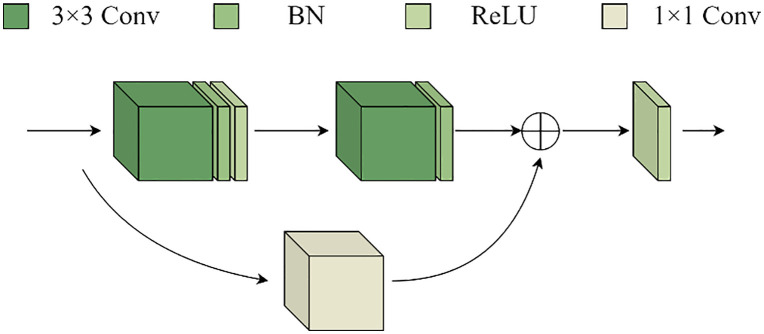
The original BasicBlock2 structure.

The U-shape structural features of the U-Net model and the concept of jumping connections serve as the foundation for the renal microvascular segmentation model known as MAAR-Net. Fig 1 illustrates the structural basis. In contrast to the conventional U-Net network model, the enhanced model adds the Depthwise Separable Channels Attention Module (DSCAM) to the BasicBlock module in the residual network, which enhances the BasicBlock and replaces the downsampling operation in the traditional U-Net model during the encoding process. The model will be designed for the Deep Separable Convolutional Attention Module (DSCAM); additionally, it will incorporate a high-feature semantic extraction layer during the coding stage, employ a 32-fold downsampling layer, and a global average pooling layer to expand the model’s sensory field and acquire richer semantic information. The jump connections contain an embedded convolutional attention module (DSCAM). To directly add the pixel values on the feature map in a point-to-point fashion during the decoding stage of the MAAR-Net model, the add method is employed in the feature map fusion; ultimately, an auxiliary segmentation branch is added to the enhanced model to further boost the model performance. For simplicity of differentiation, the BasicBlock module in the original ResNet was divided into two forms called BasicBlock1 and BasicBlock2, with their structures depicted in Figs 2 and 3.

### 3.1. Encoder

**Fig 4:**
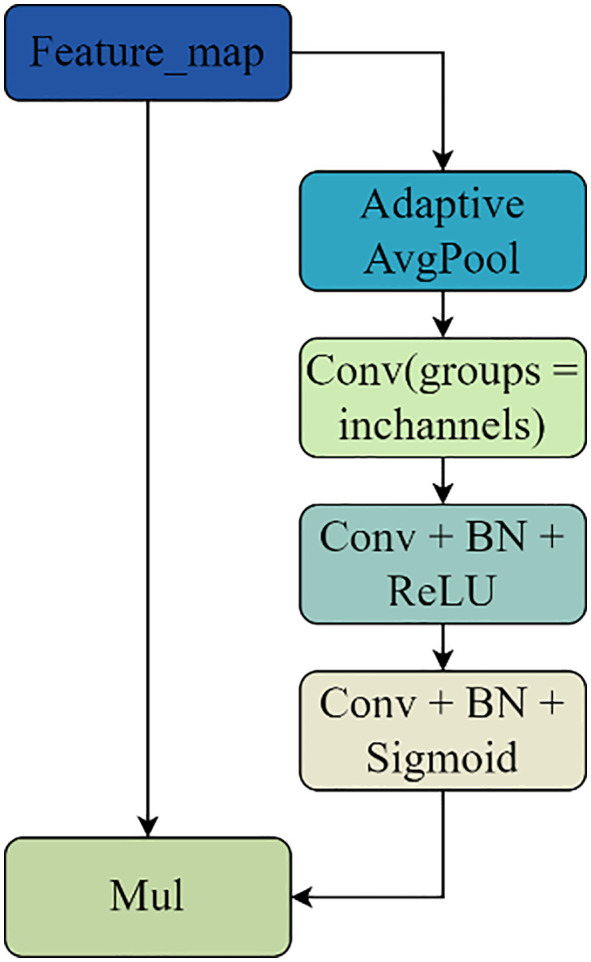
The structure of the DSCAM module.

**Fig 5:**
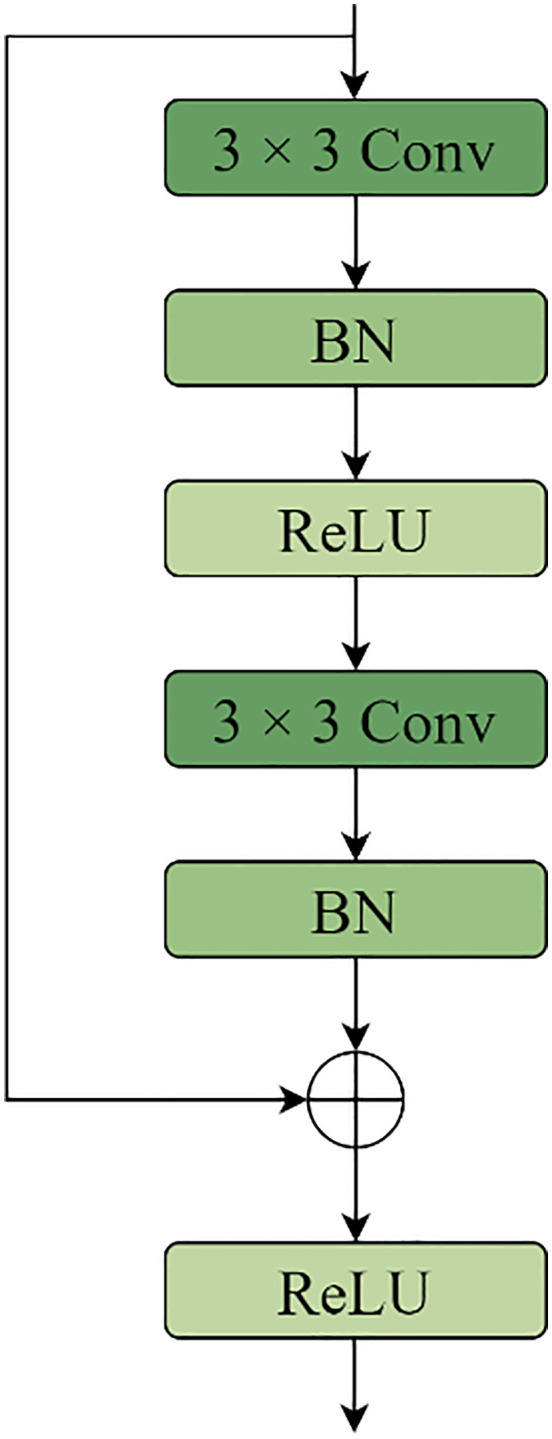
The structure of the enhanced BasicBlock1 module.

**Fig 6:**
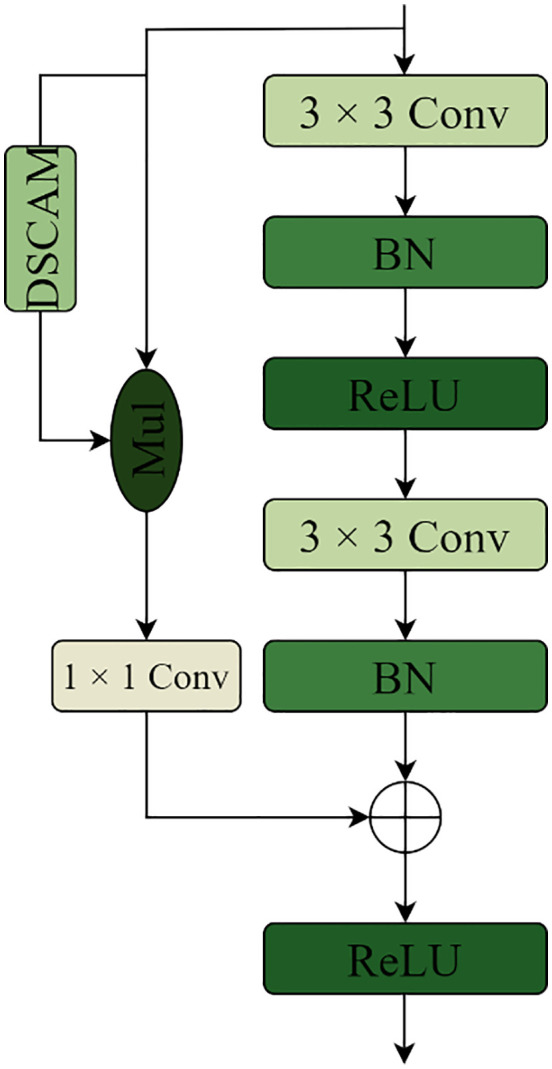
The structure of the enhanced BasicBlock2 module.

Given that the kidney contains a large number of intricately shaped microvascular structures, the improved model adds a layer of high-semantic feature information during downsampling to extract the high-semantic feature information and give the network a larger receptive field. This allows the improved model to expand its sensory field while also obtaining more semantic information about the microvascular structures in the kidney image. The network will deteriorate as the model layers deepen and the features on the feature map will become distorted as the number of convolutions increases if the convolution operation is used frequently during the encoding stage for multiple downsampling. This will cause the gradient disappearance issue to arise during the network’s backpropagation process. As a result, the BasicBlock module in ResNet is used to perform the downsampling operation during the encoding stage. The residual structure in the BasicBlock module can effectively handle the problems of gradient disappearance and network degradation. Additionally, each time a convolution operation is performed, the feature maps that are learned through the convolution operation are added together and fused with the original feature maps. This ensures that the new feature maps contain the features that were learned during the convolution operation. As a result, both the original feature map’s features and the feature map’s features following the convolution operation will be present in the new feature map. To filter the important information of the upper feature map in the channel dimension and enable the downsampling process to obtain more important information about the kidney microvascular structure, a depth-separable convolutional channel attention mechanism is added to the residual linkage based on the encoding operation using the BasicBlock module. The main function of the Depthwise Separable Convolutional Channels Attention Module (DSCAM), which is seen in Fig 4, is to create channel feature vectors to filter and extract important information from the feature map. For the input x, the DSCAM module first performs adaptive average pooling and then conducts depth-separable volume accumulation operations to obtain the channel feature vector. This is necessary because, at this point, the feature vector map in the channel dimensions exhibits a loss of correlation between the correlations; therefore, a convolution operation involving the channel correlations between the channel feature vectors must also be performed using the convolution kernel size of 1 × 1 of the ordinary convolution kernel. Finally, a batch of normalization layers and Sigmoid activation function are applied to obtain a one-dimensional vector, one-dimensional vector, one-dimensional vector, and so on. The weights on each channel are represented by the values on the one-dimensional vector; the output y is then obtained by multiplying the channel feature vectors and the input x by the dot product operation. In this attention module, the ChannelsΗ2 × 2 feature vector for x is obtained using adaptive average pooling rather than the global average pooling operation, and it is then reduced to Channels×1 × 2 by the deeply separable convolutional While the amount of parameters introduced by the depth-separable convolution operation is likewise entirely appropriate, the goal of doing this is to enhance the channel attention module’s performance. Lastly, the construction of the enhanced BasicBlock1 and BasicBlock2 is displayed in Figs 5 and 6. The depth-separable convolutional channel attention method is integrated into the residual connection of the BasicBlock1 and BasicBlock2 modules.

### 3.2. Depth separable convolutional attention module

**Fig 7:**
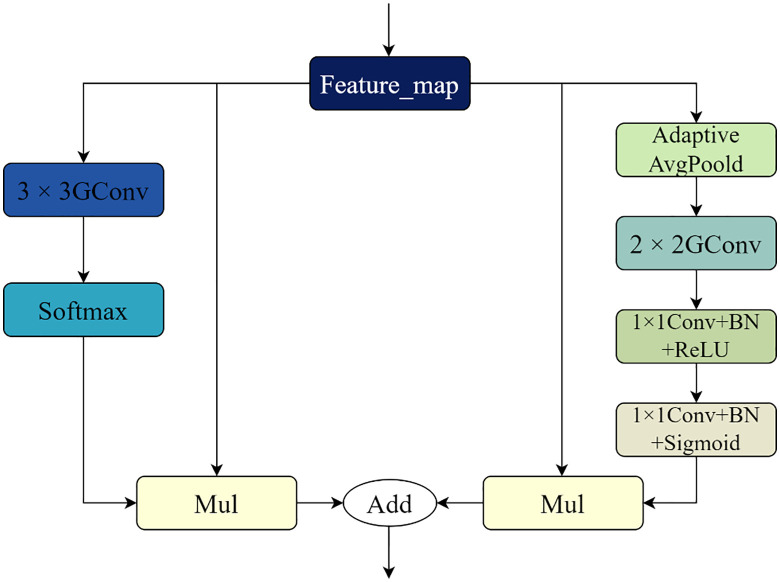
The structure of the depth-separable volume accumulation attention module.

To further increase the network’s accuracy, a growing number of academics have started to extract and filter the features from each layer of the feature map in recent years using the attention mechanism. When using a deep learning-based medical picture segmentation model, the attention mechanism can be crucial in accurately segmenting intricate features like kidney microvessels. The attention mechanism can be added to the improved segmentation model to focus on the portion of the kidney microvascular structures that are fine and have poor contrast with the surrounding tissues. Hu et al.’s proposed SE attention module [27] is a more traditional channel attention module. By using a global average pooling operation, the SE module compresses the feature map into a Channels×1 × 1 by 1 feature vector. Next, it uses a completely connected SE module that uses a global average pooling operation to compress the feature map into Channels×1 × 1 feature vectors. It then uses a fully connected operation (compressing the features first, then unfolding them; r is the compression rate) and the Sigmoid activation function to obtain the channel feature vectors with weight values ranging from 0 to 1. Finally, it uses the Scale operation to multiply each channel in the feature map by the corresponding feature vectors. In the Bisenet network, a comparable attentional process is postulated (Yu et al.) [31]. Similar to the SE module, Yu et al. have proposed an Attention Refinement Module in the Bisenet network. In contrast to the SE module, the authors substitute a convolutional layer and a batch normalization layer for the two fully connected layers. They then perform the convolutional and batch normalization operations directly on the feature vectors obtained through global average pooling to obtain the channel feature vectors. Lastly, the obtained feature vectors are multiplied by the Sigmoid function with the feature maps to realize the channel dimension’s attention mechanism. It is evident from the foregoing that the network model’s performance can be greatly enhanced by the channel attention mechanism as well as the spatial attention mechanism. Consequently, to further increase the model’s accuracy, it is imperative to investigate the most effective combination of these two techniques. The channel attention mechanism is used on the feature maps to create new feature maps, and the spatial attention mechanism is then applied to the new feature maps to provide the output results. This is how the CBAM module integrates the two processes in tandem. The original feature map can directly obtain the gradient information from both the channel and spatial directions, so improving the accuracy of the network model; the depth-separable convolutional attention module adopts the way of using the two attention mechanisms in parallel. The overall depth-separable convolutional attention module is shown in Fig 7. This allows the two attention mechanisms to work directly on the original feature map as well as in the back-propagation process of the network model. Following the two attention methods to produce two feature vector maps, the two feature vector maps execute a point-to-point summing operation. Channel superposition is not used in the intended attention module because, following superposition, a convolution operation is required to restore the number of channels, adding to the model’s computational complexity. Consequently, this combination ensures better accuracy in the model’s segmentation of renal microvascular structures while achieving an effective balance of computing effort in the model.

### 3.3. Decoder

**Fig 8:**
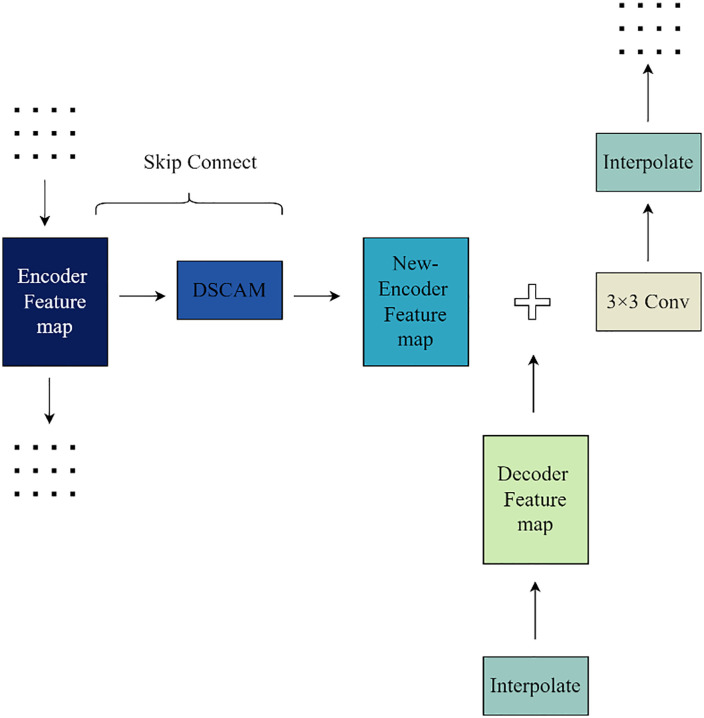
The process of upsampling.

Because of the target structure’s extreme fineness and complexity, the kidney microvascular structure segmentation task requires a lot of blank content to be filled in by the model during the decoding stage. Additionally, since learning from scratch frequently necessitates the network’s ability to guess a large amount of information, including detailed information, the network may not be able to provide a good restoration effect for some of the kidney microvascular structure’s detailed features. To achieve efficient information transfer between the encoding and decoding stages, the improved network model keeps the hopping connection design from the U-Net model. Specifically, the corresponding scale feature maps from the downsampling process are introduced into the decoding stage to fully utilize the detailed information of the renal microvascular structure found in the feature maps from the encoding stage. Furthermore, as mentioned in the previous subsection, the improved network model further enhances the effectiveness of the jump link for improving the model accuracy by embedding the depth-separable convolutional attention module designed in the previous subsection into the jump link. This allows the focus to be on the feature information of the renal microvascular structures, which are finely structured and exhibit low contrast with the surrounding tissues. During the upsampling process of the network model, there are two methods to achieve the decoding operation: directly using the upsampling function or using the deconvolution operation. The deconvolution operation is the reverse process of the ordinary convolution operation and essentially performs the convolution operation. On the other hand, the desired upsampling can be achieved by directly filling pixels with the corresponding interpolation algorithms (such as nearest neighbor interpolation, bilinear interpolation, etc.) using the upsampling function, which only requires mathematical operations. In this case, compared with the sampling function, the deconvolution operation will increase the computational load and the number of parameters of the network. Therefore, the bilinear interpolation algorithm is used in the improved model to achieve upsampling, which reduces the computational load and the number of parameters of the model while ensuring the accuracy of the network model. Compared with the sampling function, the convolution operation will add additional computational load and the number of parameters to the network. The upsampling process at a certain scale in the decoding stage of the improved model is shown in Fig 8. The operations in the figure are repeated continuously until the decoding stage is completed.

### 3.4. Auxiliary segmentation

**Fig 9:**
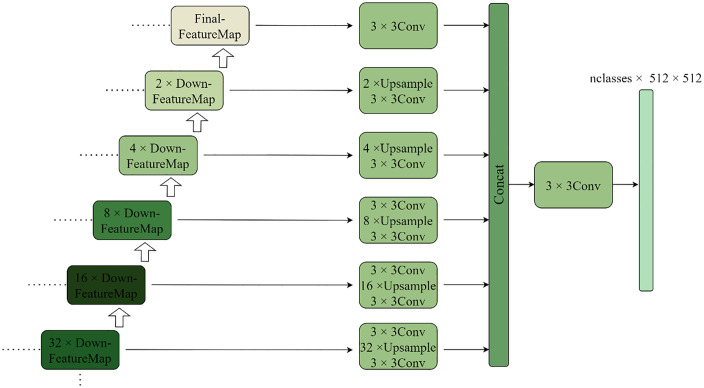
The structure of the auxiliary segmentation branch.

An auxiliary segmentation branch is also included in the modified model to further increase its efficiency in segmenting the renal microvascular systems. By enhancing the correctness of the network model, this branch task reduces computational cost throughout the model inference process. During network model training, the auxiliary segmentation branch task is turned on, and during model prediction inference, it is turned off. Furthermore, experimental evidence indicates that the auxiliary split branching task enhances the network model’s robustness. The enhanced model, which incorporates auxiliary split branching, exhibits a narrower range of fluctuation in its prediction accuracy when tested on both the validation and test sets. The network model’s decoding phase is known to progressively shrink the feature map to its initial size by repeatedly carrying out a two-fold upsampling operation. In the end, the output results are obtained, and the loss between these and the correct labels is computed. The repeated upsampling process will, however, inevitably result in the loss of some semantic information. Therefore, each layer of feature maps (which included feature maps of 1/2, 1/4, 1/8, 1/16, and 1/32 of the original size) is resized to its original size while retaining the semantic information of various sensing fields. This way, six feature maps—including the final feature map obtained in the decoding stage—are produced that are all the same size as the original map. When the feature maps are restored, the maximum multiplicity reaches 32 times the span. If these feature maps are updated with the real label loss to update the network parameters separately, it cannot effectively help to improve the network’s performance. The auxiliary segmentation branch task does not create these six maps to calculate the loss with the real label separately; instead, the information they contain is the semantic information for various sensory fields. As a result, before computing the loss using the actual labels, the six feature maps are first stacked in the channel dimension and then compressed into feature maps with classes (number of classes) using a convolution operation. By utilizing an assisted segmentation strategy, the network can integrate semantic data from many sensory fields to enhance its performance and resilience by optimizing the network model’s parameters. Fig 9 depicts the architecture of the assisted segmentation branching problem.

### 3.5. Pruning and quantification of the MAAR-Net model

When compressing the renal microvascular structure segmentation model based on the improved U-Net, two main methods were adopted: model pruning and model quantification. Model pruning can effectively remove the redundant weight data after the training of the network model is completed, thereby simplifying the model structure while retaining the model accuracy. When the model performs pruning processing, structured pruning is used, so the network model can achieve the purpose of model compression on all devices. Model quantification adopts offline quantification. By introducing quantification -de-quantification operations into the model, it can effectively reduce the model size and improve computational efficiency.

(1) Model structured pruning

Model structured pruning does not target the weight values in the network model, but rather prunes the architecture of the network model. Ultimately, the difference in accuracy between the pruned model and the original model is within a very small range. Since it is about pruning the model structure, it is crucial to determine the contribution of certain model structures in the network to the model inference results.

The renal microvessel segmentation model based on the improved U-Net is composed of a feature encoding module and a feature decoding module, and each module is made up of multiple feature layers. Therefore, when performing model pruning processing, it is necessary to reasonably select the pruned network feature layers to achieve a good effect. Secondly, the pruning processing of the improved renal microvascular structure segmentation model is mainly carried out in the channel dimension. Therefore, it is also necessary to select a reasonable method to determine the importance of each channel in the pruned feature layer. For deep learning models, the closer the changes in the network feature layers are to the model input, the greater the impact on the model results. Therefore, when selecting the pruned network feature layers, the feature layers farthest from the model input should be given priority. Secondly, when performing model pruning operations, the L2 norm is adopted to determine the importance of each channel in the pruned feature layer.

(2) Model INT8 quantification

When the model is deployed on hardware devices, due to the limited memory resources and computing power of the hardware devices, in order to further improve the performance of the model, quantification processing is also carried out on the model. To verify the effectiveness of INT8 quantification in improving model performance, the following experiments were conducted.

The model quantification adopts the static quantification method after training, that is, the offline quantification method, which is an operation that occurs after the network model training is completed. After training, static quantification needs to quantify both the model’s weights and activations. Specifically, to accelerate model inference, some layers are fused (such as fusing conv layers, relu layers, or fusing conv layers, bn layers, relu layers, etc.). Taking the fusion of conv layers and bn layers as an example, When performing fusion operations in the network, the calculation process of the bn layer is actually folded into that of the conv layer, thereby eliminating the computational overhead of the bn layer. Subsequently, quantification operations and dequantification operations are added to the network model (generally, quantification operations are added before the model input, and dequantification operations are added after the model output) to enable the network model parameters to perform inference calculations under the INT8 data type. Finally, the model is quantized and the data quantification range and scaling factor are calculated using the calibration dataset (usually the data from the training dataset), thereby maintaining the accuracy of the network and ultimately obtaining the quantized model.

## 4. Experimental results and analysis

### 4.1. Datasets and evaluation metrics

**Fig 10:**
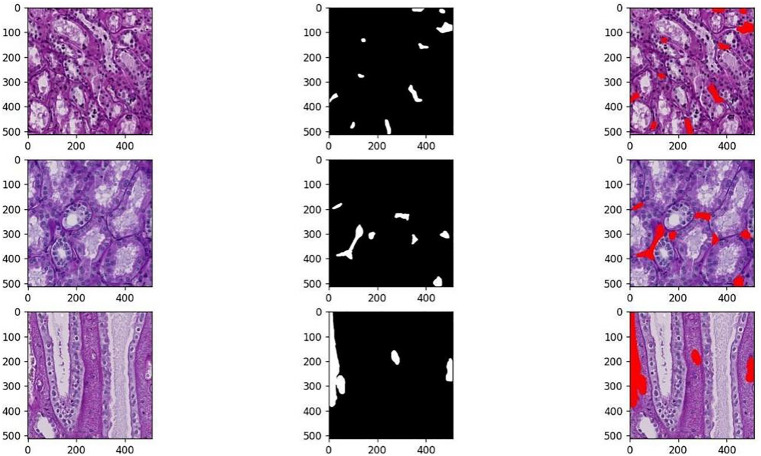
Some images of the dataset.

HuBMAP provided entire kidney 2D-PAS chromosomal tissue samples, which were used to create the dataset used in the investigations. The collection consists of segments taken from whole slide images (WSI) that have been labeled to specifically target the blood vessels, or microvascular structures, on histology slides of human kidneys. A total of 1633 RGB three-channel color images with 512 x 512 pixels make up the dataset. The dataset is divided into three sets: a training set (979 images), a testing set (327 images), and a validation set (327 images) based on a 6:2:2 ratio. Some of the images and labels are displayed in Fig 10, where the first column represents the RGB three-channel original image data, which is also the data inputted to the network model, and the second column represents the PNG format data. data, the single-channel labeled data in PNG format is shown in the second column, and the location of the kidney microvascular structure on the original image is shown in the last column.

Since the enhanced network model falls within the category of semantic segmentation, the experimental data is quantitatively analyzed using the model evaluation metrics that are frequently employed in this field, namely the F1 score and the IoU (intersection-union-ratio) score.

IoU score and F1 score are frequently used as evaluation metrics in the field of medical picture segmentation; therefore, using them to assess the renal microvascular structure segmentation model’s performance can yield accurate and dependable evaluation findings.

By computing the overlap between the predicted values and the actual labeled values of the model, the IoU score assesses the model’s performance. The interval where the IoU score is located is [0,1]. When the IoU equals 0, the predicted values and the real values completely overlap, indicating that the network’s predicted values are entirely incorrect. Conversely, when the IoU equals 1, the predicted values and the real values completely overlap, indicating that the network’s predicted values are entirely correct. The higher the IoU score, the better the network’s performance. The ratio of the intersection and union of the predicted and labeled values is the evaluation index of the IoU score., Please let mask be the set of pixels with actual labels and pred be the set of pixels that the network model predicts. The number of pixels at the intersection of pred and mask, the number of pixels in their union, the number of pixels in their union, the number of pixels in their union, the number of pixels in their union, the number of pixels in their intersection, the number of pixels in their union, and the number of pixels in their union are all considered the intersection. Equations 1–3 display the IoU score evaluation index, intersection, union, and intersection, respectively, as the sum of pixels in the intersection of pred and mask.


Inter section =| pred ∩ mask | (1)

Union =| pred ∪ mask | (2)

$$\begin{array}{c} IOU=\frac{Inter\;section}{Union} \end{array}$$ (3)

A statistical measure of a model’s precision that accounts for both recall and precision is called the F1 score. The F1 score, which has a maximum value of 1 and a minimum value of 0, is the harmonic mean of the model’s precision and recall. Better model performance is indicated by higher F1 scores. Equations 4 and 5 display the model’s recall and precision.


$$Precision\;=\frac{TP}{TP\;+\;FP}$$ (4)

$$Recall\;=\frac{TP}{TP\;+\;FN}$$ (5)

The number of pixels with a positive prediction result and a positive true label is represented by TP (True Postive), the number of pixels with a positive prediction result and a negative true label is represented by FP (False Postive), and the number of pixels with a negative prediction result and a positive true label is represented by FN (False Negative). According to the above formula, recall is the number of positive sample pixels in the prediction result as a percentage of the positive samples of the true labels, and precision is the number of positive sample pixels in the prediction result as a percentage of all the pixels predicted as positive samples. 6 displays the formula used to determine the F1 scores.


$$F1=\frac{2\times Precision\times Recall}{Precision+Recall}$$ (6)

It is evident from the calculation that a greater F1 score is only achieved when recall and precision are both higher. A model’s F1 score will be impacted if it just concentrates on improving recall and disregards precision, or vice versa. As a result, the F1 score can more accurately indicate whether or not the model performs well in positive class predictions.

### 4.2. Comparative experiment

To rigorously evaluate the efficacy of the proposed MAAR-Net, we conducted comprehensive comparative experiments against seven state-of-the-art segmentation models, including three U-Net variants (U-Net, U-Net++, U-Net3+), Deeplabv3 + , and three advanced architectures integrating attention or transformer mechanisms (TransU-Net, SwinU-Net, Attention U-Net). This benchmarking study involved eight models in total, evaluated on a dataset of 2D PAS-stained human kidney histology images, comprising 979 training, 327 validation, and 327 testing samples. All results were averaged across five independent training runs with standard deviations reported for statistical robustness.

As summarized in Table 1, MAAR-Net achieved the highest segmentation accuracy, attaining an IoU score of 0.5065 ± 0.015 and an F1-score of 0.6754 ± 0.012, outperforming all seven benchmark models. Compared to classical U-Net-based architectures, MAAR-Net demonstrated significant improvements, surpassing U-Net (IoU: 0.4768 ± 0.014), U-Net++ (IoU: 0.4815 ± 0.013), and U-Net3+ (IoU: 0.4859 ± 0.015) by 0.0297, 0.0250, and 0.0206, respectively. It also outperformed Deeplabv3+ (IoU: 0.4705 ± 0.016) by 0.0360 in IoU. Among attention-enhanced models, Attention U-Net achieved a competitive IoU of 0.4874 ± 0.015, yet MAAR-Net maintained a clear margin of 0.0191.

**Table 1:** Comparative Experiments.

Model	IoU score	Precision	Recall	F1 score	FLOPs(G)	Params(M)
U-Net	0.4768 ± 0.014	0.7215 ± 0.012	0.5897 ± 0.013	0.6486 ± 0.013	218.98	31.04
U-Net++	0.4815 ± 0.013	0.6848 ± 0.011	0.6324 ± 0.014	0.6575 ± 0.012	554.64	36.63
U-Net3+	0.4859 ± 0.015	0.6842 ± 0.013	0.6392 ± 0.012	0.6608 ± 0.014	798.97	**26.97**
Deeplabv3+	0.4705 ± 0.016	0.7003 ± 0.014	0.5926 ± 0.015	0.6421 ± 0.015	243.34	54.71
TransU-Net	0.4819 ± 0.017	0.6815 ± 0.015	0.6268 ± 0.016	0.6530 ± 0.016	392.19	105.85
SwinU-Net	0.4571 ± 0.018	0.6786 ± 0.016	0.5719 ± 0.017	0.6203 ± 0.017	300.73	61.21
Attention U-Net	0.4874 ± 0.015	0.7098 ± 0.013	0.6296 ± 0.014	0.6673 ± 0.014	249.93	34.17
MAAR-Net	**0.5065 ± 0.015**	**0.7262 ± 0.012**	0.6307 ± 0.013	**0.6754 ± 0.012**	**65.23**	28.37

Notably, TransU-Net (IoU: 0.4819 ± 0.017) and SwinU-Net (IoU: 0.4571 ± 0.018) underperformed relative to MAAR-Net. We attribute this to the limited dataset size (1,633 total samples), which may hinder transformer-based models from fully leveraging their global attention mechanisms. Transformers typically require large-scale training to model long-range dependencies effectively, whereas MAAR-Net’s architecture, integrating multi-scale attention and residual refinement, demonstrates superior adaptability to data-constrained scenarios.

In terms of computational efficiency, MAAR-Net exhibited exceptional resource efficiency. Its computational cost (FLOPs: 65.23G) was significantly lower than U-Net (218.98G), U-Net++ (554.64G), U-Net3+ (798.97G), and Deeplabv3+ (243.34G), achieving reductions of 153.75G, 489.41G, 733.74G, and 178.11G, respectively. While MAAR-Net’s parameter count (28.37M) slightly exceeded U-Net3+ (26.97M) by 1.40M, it remained notably lower than U-Net (31.04M), U-Net++ (36.63M), and Deeplabv3+ (54.71M) by 2.67M, 8.26M, and 26.34M, respectively. This balance between performance and efficiency highlights MAAR-Net’s optimized architectural design.

In conclusion, MAAR-Net achieves superior segmentation accuracy and computational efficiency, validating its practicality for renal microvascular segmentation in resource-limited settings. Its robustness against data scarcity and parameter efficiency further distinguish it as a viable solution for histopathological analysis.

**Fig 11:**
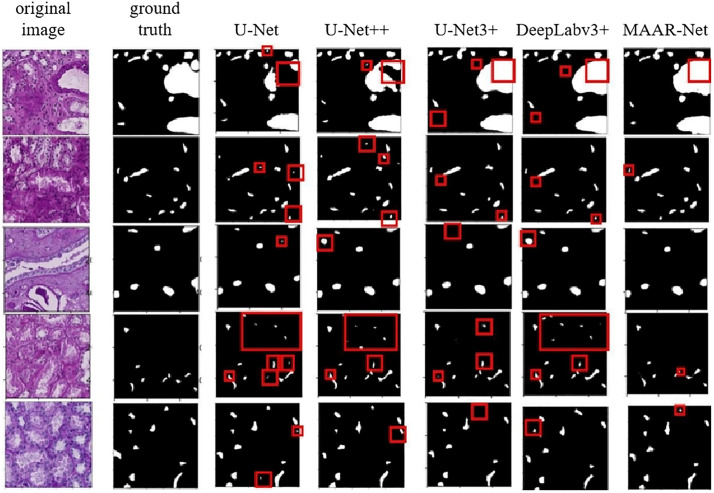
Compare the experimental results of the models.

The comparative network model and the MAAR-Net network model’s visualization findings are displayed in Fig 11. The renal microvascular structure segmentation results of the U-Net, U-Net++, U-Net3 + , TransU-Net, SwinU-Net, AttentionU-Net and DeepLabv3 + models (the areas marked by rectangles in Fig 11) show a lot of omissions and misdetections, as can be seen from the model prediction results; the segmentation results of the MAAR-Net network model in the details of the renal microvascular structure deviate from the real labeling to some extent, but the segmentation result is much better than the previously mentioned comparison network models. When compared to the previously discussed comparison network models, the MAAR-Net network model greatly reduces leakage and misdetection of renal microvascular structures, albeit having some divergence from the true labeling in terms of comprehensive information. To sum up, the MAAR-Net network model performs better at microvessel segmentation. Additionally, the model’s computational and parametric variables are kept within an optimal range, ensuring the segmentation model’s correctness, ease of use, and practicality.

### 4.3. Ablation experiments

The following ablation tests were also carried out to confirm how well each additional improvement module enhanced the improved model to segment the renal microvascular structure. The experimental results are shown in Table 2.

**Table 2:** Ablation experiment.

U-Net	U-Net encode-decode	MAAR-Net encode-decode	32 × Downsa mple&GlobalPool	DSCBAM	Auxiliary Split Branches	IoU score	Precision	Recall	F1 score	FLOPs (G)	Params (M)
√	√	–	–	–	–	0.4771	0.7208	0.5905	0.6492	218.98	31.04
√	–	√	–	–	–	0.4823	0.6720	0.6386	0.6549	61.79	13.28
√	–	√	√	–	–	0.4896	0.6533	0.6645	0.6588	64.88	25.87
√	–	√	–	√	–	0.4861	0.6846	0.6280	0.6551	62.13	13.65
√	–	√	–	–	√	0.4866	0.7068	0.6063	0.6527	61.79	13.28
√	–	√	–	√	√	0.4917	0.7074	0.6126	0.6566	62.13	13.65
√	–	√	√	–	√	0.4920	0.6677	0.6624	0.6650	64.88	25.87
√	–	√	√	√	–	0.4962	0.7064	0.6289	0.6654	65.23	28.37
√	–	√	√	√	√	0.5063	0.7267	0.6303	0.6751	65.23	28.37

Table 2 illustrates how the suggested improvement modules are permuted to incorporate several modules in the revised model and to confirm the efficacy of individual modules. To balance the performance of the network model, changes are performed for the decoding phase of the model. The U-Net network model is improved by adding many extra improvement modules before and after, which introduces more computational and parametric quantities in the model. To compare the encoding-decoding method of the improved MAAR-Net model with that of the U-Net model during the ablation experiments, the first step is to compare the two methods’ encoding-decoding techniques. Based on the results, it is evident that the encoding-decoding method of the MAAR-Net model improves the IoU score and the F1 score by 0.0052 and 0.0057, respectively, and reduces the number of parameters and computation by 157.19% and 157.19%, respectively, when compared to the original U-Net model. The enhanced encoding-decoding approach has lower computational and parametric quantities than the original U-Net model, and the segmentation accuracy of the renal microvascular structure is also increased by 157.19G and 17.76M, respectively. As a result, the MAAR-Net model’s encoding-decoding process is the main focus of the ensuing ablation experiments. The network model that combines all of the improved modules has the highest IoU score and F1 score, meaning that it currently has the highest segmentation accuracy of the renal microvascular structure, according to the evaluation indexes displayed in the table. The more improved modules used in the MAAR-Net network model, the higher the computational volume and parameter number of the model, but it also has the highest segmentation accuracy of the renal microvascular structure. Second, based on the data in the table, it can be inferred that the network model with the highest recall that combines all of the improved modules is not the best. As previously stated, recall and precision alone cannot accurately assess a model’s performance, so the F1 score serves as the primary benchmark for evaluating the model in the experiments.

**Fig 12:**
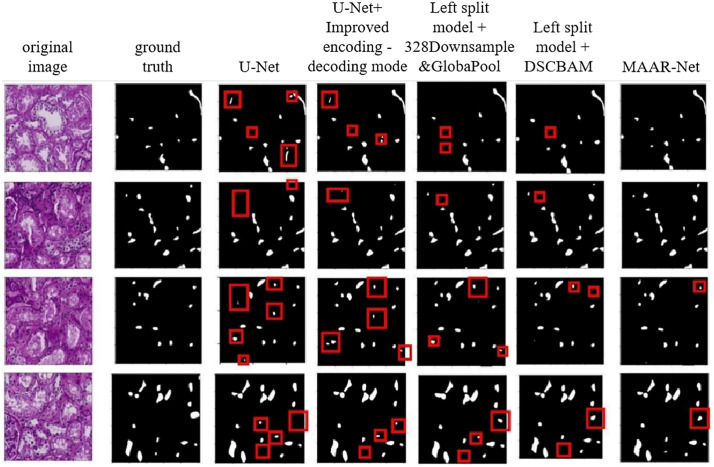
Results of the ablation experiment.

The examination of the visualization results with various enhanced modules added to the model is displayed in Fig 12. The segmentation results in Fig 12 demonstrate that, in comparison to the original U-Net model, the segmentation of renal microvascular structures using the improved encoding-decoding method has a lower leakage rate and a lower false detection rate (see, for example, the areas in Fig 12 marked by red rectangles). When the suggested improved modules are continuously added to the model, both the false detection rate and the leakage rate of renal microvascular structures show a decreasing trend. The network model provides the best segmentation effect on the renal microvascular structure and the lowest false detection and leakage rates when all the improvement modules are applied. It is evident that: every improvement module works well to enhance the kidney microvascular structure segmentation outcomes.

### 4.4. Experimental results of pruning and quantification of the model

**Fig 13:**
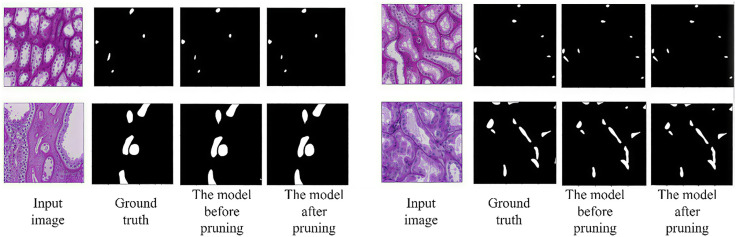
Comparison of visual results before and after pruning.

Table 3 shows the comparison of the model before and after pruning. According to the data in the table, when the model undergoes pruning and retraining, the memory space occupied by the model is reduced by approximately 35%, and the computational load and parameters are decreased by 2.0 and 8.8 respectively. Secondly, it can be seen from the visual comparison results shown in Fig 13 that the segmentation results of the renal microvascular structure of the pruned model are basically consistent with those of the model before pruning. In summary, after pruning, the accuracy of the renal microvessel segmentation model only slightly decreased, and the prediction results of the model remained basically unchanged. However, the performance of the model in terms of space occupation, computational load, and parameter quantity was significantly improved. Therefore, the model pruning operation has a positive impact on improving the overall performance of the renal microvascular structure segmentation model.

**Table 3:** Comparison of model performance before and after pruning.

Model	The memory occupied by the model	F1 score	FLOPs	Params
The model before pruning	108M	0.6751	65.23	28.37
The model after pruning	74.8M	0.6743	63.23	19.57

**Fig 14:**
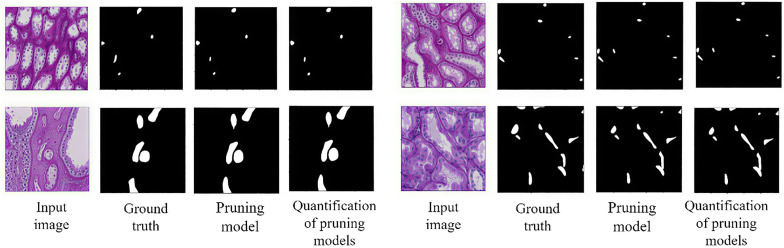
Comparison of visual results before and after pruning model quantification.

As can be seen from the data in Table 4, after performing fusion operations and INT8 quantification operations on the designated layers in the renal microvessel segmentation model, the IoU score and F1 score decreased by 0.0006 and 0.0002 respectively compared with the pruning model. However, the memory space occupied by the quantized model file is approximately reduced to one quarter of that of the pruned model file, and the inference time of the quantized model under the CPU(Intel(R) Xeon(R) CPU E5-2678 v3) is reduced by 2.4554 seconds compared with the original model. It was reduced by 1.6723 seconds compared with the pruning model. Secondly, as can be seen from the visualization comparison results shown in Fig 14, the segmentation results of the renal microvascular structure are basically the same before and after the pruning model quantification. It can be concluded from this that quantitative operations can effectively improve the performance of the renal microvascular structure segmentation model.

**Table 4:** Performance comparison after model quantification.

Model	Pruning	INT8 Quantification	The memory occupied by the model	IoU score	F1 score	Inference time
MAAR-Net	–	–	108M	0.5063	0.6751	4.3269s
MAAR-Net	√	–	74.8M	0.5052	0.6743	3.5438s
MAAR-Net	√	√	19.4M	0.5046	0.6741	1.8175s

### 4.5. Discussion and future directions

While the proposed MAAR-Net demonstrates promising performance in segmenting renal microvascular structure, several limitations warrant discussion and point to avenues for future work. Primarily, our model is currently trained and validated on 2D PAS-stained histology images. This limits its direct applicability to 3D volumetric data or other imaging modalities, which are crucial for a comprehensive assessment. The complex and variable morphology of pathological microvasculature in advanced disease stages also poses a challenge, potentially affecting segmentation consistency.

To address these limitations and further enhance performance, several advanced methodologies can be explored. First, to leverage multi-modal or multi-center data, techniques such as cross-modal alignment [39] and weighted ensemble methods [40] offer robust frameworks for fusing complementary information, which could improve the model’s generalizability and robustness against variations in staining or scanning protocols. Second, to achieve more precise and consistent boundary delineation, especially for fine and ambiguous structures, incorporating dual auxiliary information [41] mechanisms could refine feature representation by modeling complex contextual relationships. Finally, extending the current 2D framework to 3D is a critical next step. While 3D reconstruction from sparse or anisotropic slices is challenging, recent advances in automatic 3D reconstruction under constraints [42] provide valuable strategies that could be integrated to develop a true 3D segmentation pipeline, offering a more complete anatomical perspective.

## 5. Conclusion

This paper discusses the threat that kidney disorders pose to world health and the significance of comprehending the microvascular anatomy of the kidney. To aid in the identification and management of renal disorders, an enhanced U-Net model for the high-precision segmentation of renal microvascular structure is put forth. The following are some of the ways that the renal microvascular structure’s modeling has improved:

Second, to obtain a larger sensory field and more semantic information, a 32-fold downsampling layer and a global average pooling layer are added to the encoding stage of the U-Net model. A depth-separable convolutional attention module is designed and added to the model. The encoding stage of the U-Net model uses a residual structure to perform feature downsampling operations. Since the improved module adds extra memory overhead to the network, a simple and effective up-sampling method is used to balance the computational and parameter counts of the network. A 32-fold down-sampling layer and a globally averaged pooling layer are also added to the model to further improve the accuracy of the model. This module extracts and filters features from both spatial and channel dimensions. It is designed to be lighter than lightweight, so it does not bring a lot of computational overhead to the network. An additional segmentation branch is added to the model to increase its accuracy even further. This branch adds no computational or memory overhead to the model and is turned on during training and off during test inference. The accuracy of the improved MAAR-Net network model is verified through experimental data and visualization results, and it is compared with the mainstream U-Net network model and the v3 + network model in the deeplab series. Ablation studies also show the usefulness of the upgraded modules in the model and the superiority of the MAAR-Net model in segmenting the renal microvascular structure. Through the real-time test experiments of the model, the effectiveness of the renal microvessel segmentation model in practical application scenarios has been proved.

## 6. Clinical implications and future work

### 6.1. Clinical implications

The proposed MAAR-Net model, achieving superior segmentation performance on the HuBMAP dataset, offers tangible benefits for renal pathology practice and research. Firstly, the automated, high-precision delineation of microvascular structure transitions subjective histological assessment into quantifiable, objective metrics. This reduces inter-observer variability and may enable the detection of microvascular alterations indicative of early-stage renal disease, thereby enhancing diagnostic precision. Secondly, integrating this efficient model into digital pathology workflows can significantly expedite the analytical process. By providing instant preliminary segmentation, it alleviates the pathologist’s burden of manual annotation, allowing for greater focus on complex diagnostic integration and clinician consultation. Furthermore, the model’s optimization via pruning and quantification is crucial for practical deployment. It facilitates implementation on standard pathology workstation hardware, enabling real-time, on-site analysis without compromising data security through external server reliance, thus bridging the gap between algorithmic development and routine clinical utility.

### 6.2. Future work

Building upon the technical directions outlined in Section 4. 5, our future research will also pursue several practical and clinical translation goals. Primarily, expanding and diversifying the training dataset remains critical. We plan to incorporate whole-slide images (WSIs) from patients with a broader spectrum of renal pathologies and across various disease stages. Collaborating with multiple institutions to gather data from different staining protocols and slide scanners will be essential to enhance the model’s robustness and generalizability, moving it closer to clinical adoption. Secondly, and more pivotally, our focus will shift towards creating a highly efficient, deployment-optimized model for terminal-side inference. Building on the current pruning and quantification efforts, we will explore advanced model compression techniques, such as neural architecture search (NAS) tailored for low-resource environments, and knowledge distillation to develop an ultra-lightweight yet accurate variant of MAAR-Net. The ultimate goal is to achieve real-time segmentation on portable diagnostic devices or standard pathology workstation hardware without internet dependency. This edge-deployment strategy will not only safeguard patient data privacy by eliminating the need for cloud transmission but also significantly increase the accessibility and practicality of AI-assisted pathology in diverse healthcare settings, including resource-constrained laboratories. Finally, to translate technical performance into clinical utility, we will develop an interactive, clinician-in-the-loop software interface that integrates the optimized model. This tool will allow pathologists to effortlessly verify, correct, and query model outputs within their existing digital workflow, thereby facilitating seamless adoption and providing immediate decision support at the point of care.
